# Circ-EnviroPredict: A machine learning-based tool to predict potential involvement of circRNAs with cold and drought stress through a Word2Vec approach

**DOI:** 10.1371/journal.pone.0350943

**Published:** 2026-06-18

**Authors:** Maria Clara Martins Ferreira, Frederico Schmitt Kremer, Vanessa Galli

**Affiliations:** Technological Development Center, Division of Biotechnology, Federal University of Pelotas, Pelotas, RS, Brazil; Shandong Agricultural University, CHINA

## Abstract

Circular RNAs (circRNAs) are a class of RNAs characterized by a covalently closed loop structure formed between the 5’ and 3’ splice sites. Over the years, circRNAs have been shown to act in post-transcriptional regulation and as potential sponges for miRNAs. Although the number of circRNAs identified has grown and several databases have been developed, challenges persist in analyzing large datasets and extracting molecular information. Machine learning-based tools have been gaining attention due to their potential to massively analyze molecules and understand patterns in response to stresses. However, there is still a lack of studies that connect circRNAs and abiotic stress using this approach. In this study, we developed circ-EnviroPredict, a tool designed to predict the potential involvement of circRNAs in cold and drought stress conditions based on biological sequence data. Using a Random Forest–based methodology, the circ-EnviroPredict tool was trained using rice and maize circRNA sequence data, in which k-mers were transformed into vector representations using the Word2Vec approach. Using independent test sets, it was possible to obtain accuracy values ~77% and ~81% forcold and drought models respectively. It was also possible to validate using data from other plant species, including *Arabidopsis thaliana, Glycine max and Triticum aestivum*. In addition, a k-mer density analysis revealed an enrichment of AT-rich motifs in circRNAs associated with abiotic stress conditions, providing biological insights into the sequence patterns captured by the predictive models. These results provide insights into how machine learning and Word2Vec techniques can be used to classify the potential involvement of plant circRNAs under abiotic stress conditions using biological sequence data.

## Introduction

Circular RNAs belong to a class of endogenous noncoding RNAs whose structure forms a covalently closed loop between the 5’ and 3’ splice sites through a non-canonical back-splicing event [1]. Circular RNAs can originate from one or more exons, from introns, from both exons and introns, or from intergenic regions. Most circRNAs are derived from one or more exons of protein-coding genes known as parental genes [2]. Furthermore, circRNAs are conserved and widely distributed in a variety of eukaryotic organisms, including mammals and plants [3]. More than 700 orthologous gene pairs that produce circRNAs were found between *Arabidopsis* and rice, demonstrating that circRNAs are also widely conserved among different plant species [4]. Over the past few years, a significant number of circRNAs have been identified in different plant species, such as rice [4], soybean [5], wheat [6], tomato [7] and maize [8]. This has also led also to the creation of public databases, such as CropCircDB [9], PlantCircNet [10] and PlantcircBase [11].

Regarding their biological role, it has been demonstrated that circRNAs can act as possible miRNA sponges [4], interacting with RNA polymerase II or other RNA-binding proteins (RBPs) [12] and even in the negative modulation of the expression of their parental genes [13]. Considering the importance of these molecules in regulatory biological processes, several research groups have increasingly published studies to elucidate the role and expression of circRNAs in plants under environmental stress conditions [6,14,15], since such conditions lead to loss of productivity and economic losses.

Recently, in 2024, a whole-transcriptome study aimed at identifying endogenous competing RNA (ceRNA) networks related to cold tolerance in *japonica* rice varieties was published [16]. In this study, the researchers identified 364 circRNAs that showed significant differential expression under low-temperature stress conditions, 224 miRNAs and 12183 differentially expressed mRNAs. The WRKY family was the most prominent transcription factor family involved in cold tolerance [16]. In addition to cold stress caused by low temperatures, another important factor that negatively affects plant growth and development is drought stress. Yin et al. conducted a study using transgenic plants overexpressing ath-circ032768 together with an STTM-miR472 silencing vector [17]. In this work, it was demonstrated that ath-circ032768 was able to competitively inhibit the degradation of the RPS5 gene (related to a stress resistance protein) by miR472 through a ceRNA network. This ath-circ032768–miR472-RPS5 mechanism resulted in an increased expression of DREB2A, RD29A and RD29B genes, improving the drought tolerance response of plants [17].

Along with advances in bioinformatics and sequencing techniques, and despite the growing understanding of circRNA roles in these stress conditions, many challenges have emerged over the years, such as analysis of massive datasets and challenges in mining and extracting new information about molecular characteristics of biological sequences. In this context, machine learning-based tools are gaining more and more attention in published bioinformatics articles [18,19], being recognized as a high-performance and scalable system for data-driven discoveries [20]. However, to date, few studies have been published relating circRNA data to abiotic stress in plants using machine learning-based approaches. Most studies in this area focus on the identification of circRNAs [21,22] or on studies involving abiotic stress, but with miRNA molecules [23,24]. To date, only one article has been published correlating circRNA data focused on abiotic stress prediction by machine learning [25]. In this article, researchers developed machine learning models using K-tuple nucleotide (KNC) and Pseudo-KNC (PKNC) features to numerically represent circRNAs and predict the involvement of these molecules with abiotic stress [25].

Laboratory experimental methods are expensive and time-consuming. Therefore, it is very important to develop new computational methods capable of assisting experimental studies. Since machine learning models learn from available data and identify patterns that can be applied to previously unseen data, such approaches present strong potential for the development of bioinformatics tools. In addition, the approach proposed here can be a good alternative when compared to alignment-based tools, for example, which depend on biological reference sequences that are already available.

Therefore, our objective in this study was to develop a machine learning-based tool to predict the potential involvement of circRNAs in cold and drought stress using features derived from the circRNA biological sequences. We were able to achieve this goal through the development of an innovative methodology based on k-mers (substrings of length k contained in a nucleotide sequence) segmentation and Word2Vec (a neural network-based embedding technique).

## Materials and methods

### Obtaining raw data

We collected circRNA data of *Oryza sativa* (rice) and *Zea mays* (maize) from two public databases: CropCircDB (http://deepbiology.cn/crop/index.php/Home/Html/Download) and PlantcircBase (http://ibi.zju.edu.cn/plantcircbase/download.php). The distribution of environmental condition labels among the 63,048 rice circRNAs obtained from CropCircDB was as follows: 50,187 circRNAs with control label, 5,724 with cold stress label and 824 with drought stress label. For the maize circRNA data, 20,809 circRNAs were labeled as control and 11,206 had the drought stress label. For this work, only circRNA sequences associated with control conditions, cold stress and drought stress were used. Regarding the PlantcircBase data, genomic sequences of 10,381 maize circRNAs and 43,883 rice circRNAs were used. In summary, circRNA sequence data obtained by CropCircDB were used to train and test the models (since this data had environmental condition labels) and the PlantcircBase data were used as the corpus to build the Word2Vec vocabulary.

### Feature engineering using Word2Vec approach

The Word2Vec approach was implemented for feature engineering. Word2Vec is a neural network-based word embedding technique known for its ability to map words into a high-dimensional vector space. This method assigns a fixed-length real-value vector V(m)∈ R^m^ to any word (w) in a dictionary, where V(m) represents the word vector of w and m is the length of the word vector. This technique ensures that words sharing similar semantics are located closer within the vector space. The method takes a text corpus as input and produces vector representations as output. For this work, the Word2Vec technique was applied using the Gensim library (https://pypi.org/project/gensim/). The embeddings were generated using the Continuous Bag-of-Words (CBOW) architecture, with a window size of 5, minimum word frequency of 5, and vector dimensions of 64 and 100. The model was trained for one epoch, and other parameters were kept as default, including negative sampling (negative = 5). The text corpus used to construct the vocabulary consisted of circRNA sequence data from rice and maize obtained from the PlantCircBase database. The Biopython library was used to read the files in FASTA format.

In addition, different k-mer sizes (3-mers, 4-mers and 5-mers) and vector dimensions (64 and 100) were evaluated. The circRNA sequences obtained from CropCircDB were segmented into substrings (k-mers) and then transformed into vectors through trained Word2Vec models. The vector representation process with Word2Vec generates a vector for each identified k-mer with a predefined vector size (for this work, sizes of 64 and 100 were tested). This means that for each k-mer, the Word2Vec model generates a vector of 64 or 100 dimensions. Finaly, the features representation corresponds to the sum of the vectors for all k-mers found in the sequence. This process of generating the datasets was implemented in Python.

Since the data across environmental conditions were imbalanced, the imbalanced-learn library (https://imbalanced-learn.org/stable/) was used. For the development of machine learning models focused on predicting the potential involvement of circRNAs with the cold stress condition, only rice circRNA data were used to train the models. In this scenario, an approach based on RandomUnderSampler was used in control-condition samples, where the final amount of circRNA data from the control condition decreased from 50187 to 8177, to achieve a more balanced dataset with the number of samples labeled with “cold stress” (5724 circRNAs). For the development of machine learning models focused on predicting the potential involvement with the drought stress, maize and rice circRNA data were used to generate features and train the models. Since the number of circRNAs belonging to the control group was much larger than the number of circRNAs labeled with the drought stress condition in this scenario too, it was necessary to use a combined approach of RandomUnderSampler and RandomOverSampler techniques to balance the data. After using this approach, the total amount of circRNA data for training was 34886 (17443 were for the control condition and 17443 for the drought stress condition).

For binary classification (supervised machine learning approach), the target variable for training the classification models was assigned to the “stress” column, where the name of the environmental condition was converted to a numeric variable: 0 for control situation (without any abiotic stress) and 1 for a specific abiotic stress condition (cold stress or drought stress depending on the model).

Eight feature sets were generated for training and testing the models: feature set 1A (containing vector data of size 64 based on k-mers of length 3-mers as input variables and cold stress column as the target variable); feature set 2A (containing vector data of size 100 based on k-mers of length 3-mers as input variables and cold stress column as the target variable); feature set 3A (containing vector data of size 64 based on k-mers of length 4-mers as input variables and cold stress column as the target variable); feature set 4A (containing vector data of size 100 based on k-mers of length 4-mers as input variables and cold stress column as the target variable); feature set 1B (containing vector data of size 64 based on k-mers of length 3-mers as input variables and drought stress column as the target variable); feature set 2B (containing vector data of size 100 based on k-mers of length 3-mers as input variables and drought stress column as the target variable); feature set 3B (containing vector data of size 64 based on k-mers of length 4-mers as input variables and drought stress column as the target variable); feature set 4B (containing vector data of size 100 based on k-mers of length 4-mers as input variables and drought stress column as the target variable).

### Machine learning models

Python was used for the development of machine learning models and scikit-learn library (https://scikit-learn.org/) was the main library used for training, testing and evaluating the supervised machine learning models. In addition, for clustering techniques, algorithms implemented in scikit-learn were used, such as: t-SNE (https://scikit-learn.org/stable/modules/generated/sklearn.manifold.TSNE.html). T-SNE (T-distributed Stochastic Neighbor Embedding) is a statistical method for visualizing high-dimensional data, providing each data point with a location on a two or three-dimensional map, converting similarities between data points into joint probabilities.

For supervised machine learning-based models, the data were processed in the feature engineering stage and subsequently randomly split into training and test sets using the “train_test_split” module of the scikit-learn library. For testing and model evaluation, 80% of the data were used to train the models and 20% of the data were allocated to the test set. In addition, different supervised classification machine learning algorithms were tested.

The metrics used to evaluate the different machine learning algorithms were: accuracy, precision, recall, f1-score, AUC – ROC and AUPRC (area under the precision-recall curve). Accuracy represents the ratio between the number of correct predictions and the total number of predictions. The f1-score metric corresponds to the harmonic mean of precision (a measure of the number of correctly identified positive cases out of all predicted positive cases) and recall (a measure of the number of correctly identified positive cases out of all real positive cases). The AUC – ROC curve is a performance measure for classification problems at various threshold settings. ROC is a probability curve, and AUC represents the degree or measure of separability. It indicates how well the model can distinguish between classes. The higher the AUC, the better the model is at predicting classes 0 from class 1. AUPRC summarizes the trade-off between Precision (Positive Predictive Value) and Recall (Sensitivity) by calculating the integral of the Precision-Recall curve, providing a more robust evaluation in scenarios involving significant class imbalance. DummyClassifier algorithm was used as a comparison algorithm, because this algorithm makes predictions that ignore the input features. In this sense, it serves as a simple baseline to compare with other more complex classifiers. The other models should outperform DummyClassifier, to conclude that the model was learning the underlying patterns, and not just making random predictions. Finally, the best models (the models that presented the best performance) were saved using the joblib library (https://joblib.readthedocs.io/en/stable/generated/joblib.dump.html). To ensure a fair and reproducible benchmark, all machine learning models, including the Random Forest classifier, were implemented using their default hyperparameter configurations as provided by the Scikit-Learn (v1.3.2) library. Specifically, the Random Forest was grown with 100 decision trees based on the Gini impurity criterion.

### Approximate nearest neighbors analysis

To better understand the similarity between circRNA sequences in different biological conditions and which patterns the algorithms were learning, a nearest neighbor search analysis was performed using Python and the Annoy (Approximate Nearest Neighbors Oh Yeah) technique. Annoy (https://github.com/spotify/annoy) is a library that allows efficient identification of nearest neighbors in large datasets, particularly in applications involving vectors, such as machine learning and information retrieval. With this technique, multiple search trees are constructed, where each tree is based on a random sample of data. The idea is to partition the space into regions, allowing for faster neighbor searches. In this sense, an analysis using Annoy library was performed to search for the 5 nearest neighbors of circRNA sequences from the group linked to the abiotic stress situation. To perform this analysis, the following datasets were generated:

- Word2vec vector data of 22412 maize circRNAs were divided equally to generate two groups: a “control” group containing 11206 circRNAs linked to the control condition and 11206 circRNAs linked to the drought stress condition.
- Word2vec vector data of 11448 rice circRNAs were equally divided to generate two groups: “control” group containing 5724 circRNAs linked to the control condition and 5724 circRNAs linked to the cold stress condition.

After that, a Python script was developed using the Annoy library in which each circRNA belonging to a specific stress condition was sequentially analyzed and compared to sequences from control and stress conditions. The aim was to compare circRNA sequences belonging to a specific abiotic stress condition and determine whether greater similarity existed with sequences from the stress group or if there were more similarities with sequences from the control group. For each stress sequence, the script searched for the 5 closest neighbors. Self-matches were ignored, ensuring that only unique neighbors were counted. To generate this analysis, maize and rice circRNA vector data generated by Word2Vec were added to an index, where each vector represents a numerical representation of an item. The Annoy library then builds several search trees, where each tree is built based on a random sample of the data. When querying the data, Annoy analyzes the trees to find the closest neighbors using approximation.

### k-mer density analysis

To complement the Approximate Nearest Neighbors analysis, further characterize the sequence signature of stress-responsive circRNAs, and improve the interpretability of the machine learning models, a k-mer density analysis was performed. Genomic sequences of all circRNAs available in the dataset (control, cold stress, and drought stress groups) were decomposed into 3-mer units.

To mitigate bias caused by variation in sequence length, k-mer counts were normalized by their density per 1,000 nucleotides. Density was calculated according to the following formula:


$$\begin{array}{c} D_{i,j}=\left(\frac{C_{i,j}}{L_{j}}\right)\times \text{1}\text{0}\text{0}\text{0} \end{array}$$

Where:

- C_i,j_ represents the absolute count of the 3-mer i in sequence j;
- L_j_ represents the length of sequence j in nucleotides;
- 1000 is a scaling factor used to express the density as occurrences per kilobase (kb).

To identify changes in genomic signatures between experimental conditions, the density delta (ΔD) was calculated. This value represents the arithmetic difference between the mean densities of the stress and control groups. A positive delta (Δ) indicates that the motif is enriched under stress conditions, while a negative value indicates enrichment in the control condition.


$$\begin{array}{c} \Delta D_{i}=\overset{―}{D_{i,Stress}}-\overset{―}{D_{i,Control}} \end{array}$$

Additionally, the proportional shift in motif density between stress and control groups was evaluated using Fold-change (FC) and Log2 Fold-change (Log2FC) analyses. A small constant (ϵ = 0.01) was added as a pseudocount to both the numerator and denominator to ensure numerical stability and avoid division by zero in motifs with very low density.

The formulas used were:


$$\begin{array}{c} FC_{i}=\frac{\overset{―}{D_{i,Stress}}+\varepsilon }{\overset{―}{D_{i,Control}}+\varepsilon } \end{array}$$

$$\begin{array}{c} Log_{\text{2}}FC_{i}={\text{log}}_{\text{2}}\left(\frac{\overset{―}{D_{i,Stress}}+\varepsilon }{\overset{―}{D_{i,Control}}+\varepsilon }\right) \end{array}$$

Where $\overset{―}{D_{i,Stress}}$and $\overset{―}{D_{i,Control}}$ represent the mean density of motif iii in the stress and control experimental groups, respectively.

These analyses allowed the identification of sequence motifs that are preferentially enriched or depleted under abiotic stress conditions, providing additional biological insight into the sequence patterns captured by the machine learning models.

### circ-EnviroPredict tool development

The circ-EnviroPredict bioinformatics tool was developed using the Streamlit library (https://streamlit.io/). The Streamlit library is capable of transforming Python scripts into web applications. To develop the application, the best-performing models trained to predict involvement with cold and drought stress were chosen and incorporated into the reference file (app.py). Additionally, checks and tests were implemented, such as, to check if the input sequence is valid and unit tests.

## Results

### Supervised approach – Evaluation of cold stress models

Using k-mer (k = 3, 4 and 5) vector representations of rice circRNAs sequences, different supervised classification algorithms were evaluated to develop prediction models for circRNAs involvement in cold stress conditions. Although k-mer lengths up to k = 5 were evaluated, no performance improvement was observed beyond k = 4, as shown in S1 Table. In addition, increasing k-mer length resulted in longer processing and training times due to the higher dimensionality of the feature space. Therefore, subsequent analyses were focused on k = 3 and k = 4, with k = 3 selected for the final models due to its favorable balance between predictive performance and computational efficiency. The models were trained with four sets of features: Sets 1A, 2A, 3A and 4A (described in Materials and Methods section). The models were evaluated using an independent test dataset, and the top three models are described in Table 1. A DummyClassifier was used as a baseline model for comparison.

**Table 1:** Evaluation of machine learning algorithms used to develop models focused on predicting the involvement of circRNAs and cold stress.

Feature set	ML algorithm	Accuracy	Precision	Recall	F1-score
1A (3-mers, vector size: 64, target: control vs cold)	RandomForestClassifier	0.77	0.76	0.75	0.75
	LGBMClassifier	0.75	0.74	0.73	0.73
	ExtraTreesClassifier	0.74	0.74	0.72	0.72
	XGBClassifier	0.72	0.71	0.71	0.71
	DummyClassifier	0.59	0.30	0.50	0.37
2A (3-mers, vector size: 100, target: control vs cold)	ExtraTreesClassifier	0.75	0.74	0.73	0.73
	RandomForestClassifier	0.74	0.73	0.72	0.73
	LGBMClassifier	0.74	0.73	0.72	0.73
	XGBClassifier	0.72	0.71	0.70	0.71
	DummyClassifier	0.59	0.30	0.50	0.37
3A (4-mers, vector size: 64, target: control vs cold)	LGBMClassifier	0.72	0.71	0.70	0.71
	RandomForestClassifier	0.72	0.71	0.70	0.70
	ExtraTreesClassifier	0.72	0.71	0.70	0.70
	XGBClassifier	0.71	0.69	0.69	0.69
	DummyClassifier	0.59	0.30	0.50	0.37
4A (4-mers, vector size: 100, target: control vs cold)	LGBMClassifier	0.73	0.72	0.72	0.72
	RandomForestClassifier	0.73	0.72	0.71	0.71
	ExtraTreesClassifier	0.72	0.71	0.70	0.70
	XGBClassifier	0.71	0.70	0.70	0.70
	DummyClassifier	0.59	0.30	0.50	0.37

**Fig 1:**
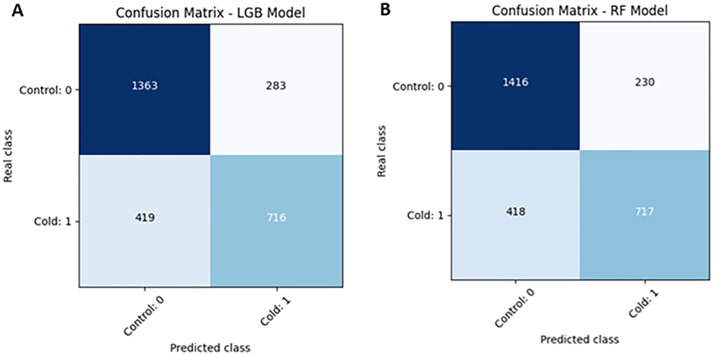
Confusion matrix generated from cold stress models using Word2Vec data (3-mers and vector size 64). **(A)** LGBMClassifier. **(B)** Random Forest.

In general, tree-based algorithms achieved the best performance metrics (Table 1), particularly RandomForestClassifier, LGBMClassifier and ExtraTreesClassifier. When comparing models trained with vectors generated from 3-mers and 4-mers, the metrics were very similar, but in some cases the 3-mers models presented better metrics. Furthermore, increasing the number of features (64 versus 100) does not seem to have any significant impact. To understand the patterns of success in cold stress involvement classification models, a confusion matrix was generated for models trained using LGBMClassifier and Random Forest using the Word2Vec vectors generated from k-mers of 3-mers and vector size 64 (the models that achieved the best performance metrics) (Fig 1).

As can be seen in Fig 1, the LGBMClassifier and Random Forest algorithms produced very similar prediction patterns. These models produced errors in approximately 15% of the cases where the model predicted the control condition for a sequence that actually belonged to the cold stress condition. Approximately 10% of errors occurred when the model predicted a cold stress condition for the sequence but in fact that sequence belonged to the control group. One of the differences between these two algorithms is that Random Forest builds multiple decision trees during training and combines their outputs through voting or averaging, while LightGBM is a boosting algorithm that builds decision trees sequentially, where each new tree corrects the errors of the previous one.

**Fig 2:**
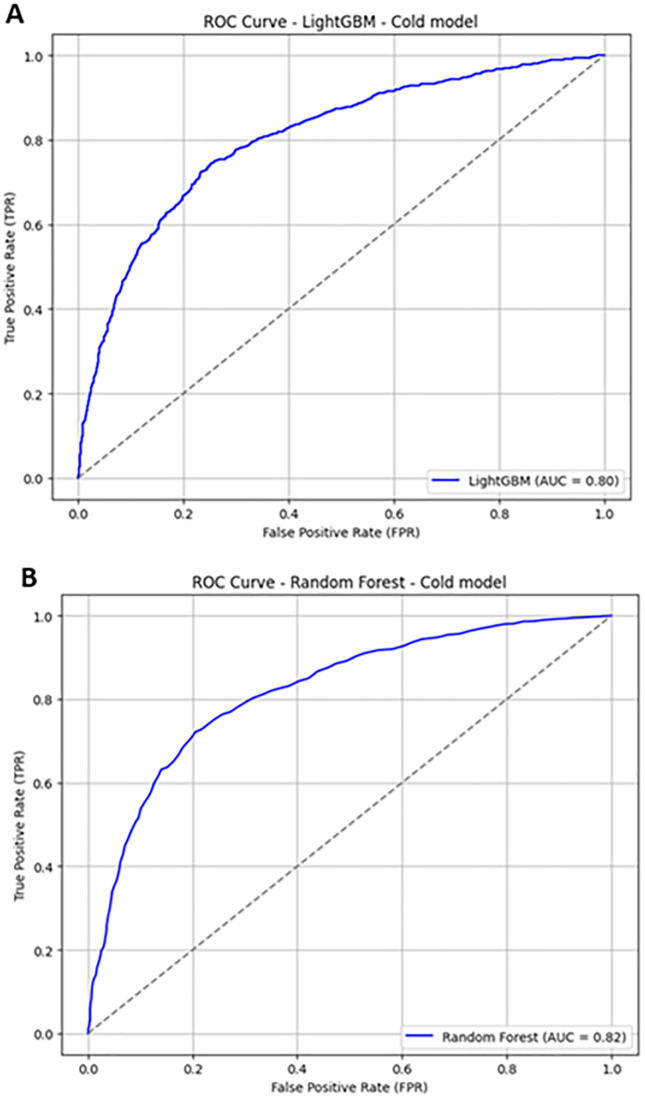
AU-ROC curve for evaluating the performance of cold stress models. **(A)** LGBMClassifier. **(B)** Random Forest.

To complement the generation of model evaluation metrics, an AU-ROC curve analysis was also performed with Random Forest and LGBM models trained with the same feature set (Fig 2), where ROC curve represents the relationship between the true positive rate and the false positive rate, and AUC represents the degree or measure of separability. It indicates how well the model can distinguish between classes.

In Fig 2, we can see that both models had AU-ROC values equal to or above 0.80, but Random Forest has a slight advantage (0.82) in terms of discrimination between classes. This means that Random Forest has a slightly better rate of identifying positive examples without generating too many false positives. In addition to AU-ROC, we also evaluated model performance using AUPRC, which is particularly informative for imbalanced datasets. The results showed that Random Forest consistently outperformed LightGBM. Random Forest achieved an AUPRC of 0.7574, while LightGBM reached 0.7368. This result further supports the robustness of the Random Forest model, especially in capturing true positive predictions under class imbalance scenarios.

In addition, a cross-validation analysis was also performed to evaluate machine learning models from the Word2Vec 3-mers set. Cross-validation helps assess model generalization, that is, to identify whether a model performs well on a training set but poorly on a validation set, which can lead to overfitting. The 4-fold cross-validation analysis was generated for the Random Forest and LightGBM algorithm models, using the K-Fold Cross-Validation approach, where the scores obtained were:

- LightGBM: Scores – [0.7013809, 0.70589928, 0.70359712, 0.72143885]. Average of 0.71.
- Random Forest: Scores – [0.71375144, 0.70935252, 0.70273381, 0.71856115]. Average of 0.71.

The prediction accuracy of the independent test set was similar to that of the cross-validation, which showed that the developed models were not strongly affected by overfitting or underfitting.

### Supervised approach – Evaluation of drought stress models

To improve model performance, the drought stress prediction models were trained using circRNA data from both rice and maize, unlike the approach focused on cold stress, which used only rice circRNA data. The difference here is that there are samples labeled with drought stress condition in both plant species. Thus, new datasets were created by combining rice and maize circRNA data. The advantage of combining data from different plant species, besides providing more data for the model, is the possibility of transferring learned patterns to new plant species and being able to perform the prediction using circRNA data from any species as input. The models obtained from classification algorithms (trained with feature sets 1B, 2B, 3B and 4B – described in Materials and Methods), focus on the prediction of circRNA involvement with drought stress are described in Table 2.

**Table 2:** Evaluation of machine learning algorithms used to develop models focused on predicting the involvement of circRNAs and drought stress.

Feature set	ML algorithm	Accuracy	Precision	Recall	F1-score
1B (3-mers, vector size: 64, target: control vs drought)	LGBMClassifier	0.82	0.83	0.82	0.82
	RandomForestClassifier	0.81	0.81	0.81	0.81
	ExtraTreesClassifier	0.80	0.80	0.80	0.80
	XGBClassifier	0.80	0.80	0.80	0.80
	DummyClassifier	0.50	0.25	0.50	0.33
2B (3-mers, vector size: 100, target: control vs drought)	LGBMClassifier	0.82	0.84	0.82	0.82
	RandomForestClassifier	0.81	0.81	0.81	0.81
	XGBClassifier	0.80	0.80	0.80	0.80
	ExtraTreesClassifier	0.79	0.79	0.79	0.79
	DummyClassifier	0.50	0.25	0.50	0.33
3B (4-mers, vector size: 64, target: control vs drought)	LGBMClassifier	0.82	0.83	0.82	0.81
	RandomForestClassifier	0.80	0.80	0.80	0.80
	XGBClassifier	0.80	0.80	0.80	0.80
	ExtraTreesClassifier	0.79	0.79	0.79	0.79
	DummyClassifier	0.50	0.25	0.50	0.33
4B (4-mers, vector size: 100, target: control vs drought)	LGBMClassifier	0.82	0.83	0.82	0.82
	RandomForestClassifier	0.81	0.81	0.81	0.81
	ExtraTreesClassifier	0.79	0.79	0.79	0.79
	XGBClassifier	0.79	0.79	0.79	0.79
	DummyClassifier	0.50	0.25	0.50	0.33

As can be seen in Table 2, drought stress models combining rice and maize circRNA sequence data in the training stage had a significant increase in metrics when compared to cold stress models (Table 1). In all feature sets, it was possible to obtain accuracy and f1-score values around 80%. This demonstrates that the Word2Vec approach can be a good proposal for a methodology for prediction using only circRNA sequence data and that the increase in the amount of data contributed to a better understanding of the patterns by the algorithms. In addition, once again, the classification algorithms with the best evaluation metrics were LGBMClassifier and RandomForestClassifier, confirming that tree-based models are apparently the best choice for this type of application.

Another point is that models trained with vector data based on 4-mers presented similar metrics compared to models trained with 3-mers, but the training time of the models was much longer when using the feature set of 4-mer and vector size of 100. Therefore, aiming at optimizing machine processing capacity and execution speed of the data preprocessing and model prediction steps, especially if the model receives more data from other plant species in the future, the set of features generated from 3-mer approach with 64 features continues to be the best option, as previously observed in cold stress models.

**Fig 3:**
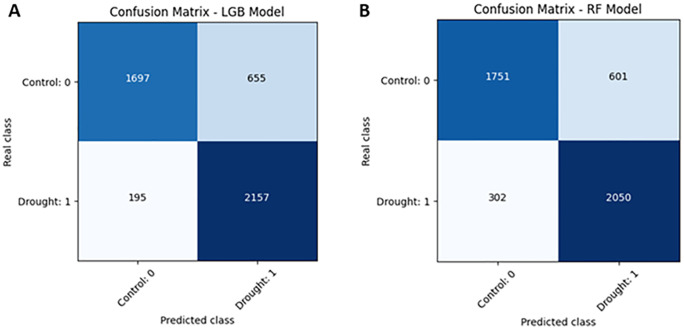
Confusion matrix generated from drought stress models using Word2Vec data (3-mers and vector size 64). **(A)** LGBMClassifier. **(B)** Random Forest.

To understand where models focused on drought stress were making right and wrong predictions, a confusion matrix was generated using the LightGBM and Random Forest algorithms and the 1B feature set, as shown in Fig 3.

**Fig 4:**
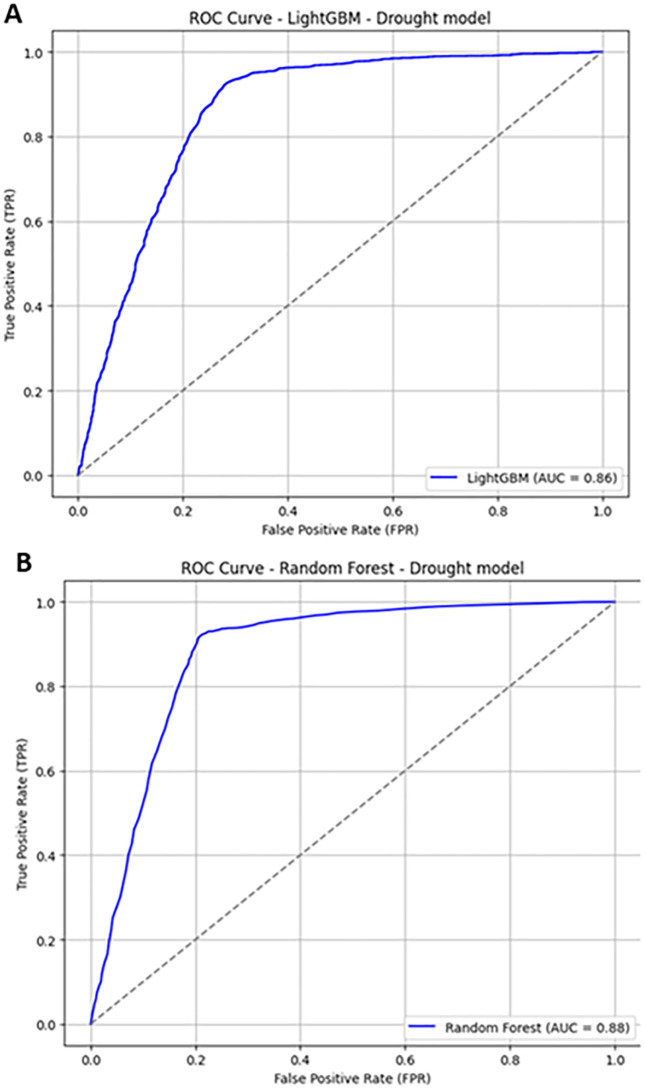
AU-ROC curve for evaluating the performance of drought stress models. **(A)** LGBMClassifier. **(B)** Random Forest.

We can observe in Fig 3 that only ~900 circRNAs out of a total of 4704 in the test sample were incorrectly predicted in both the LGBMClassifier and Random Forest algorithms. This information confirms that data increment can help improve the accuracy and sensitivity of the model. We also repeated the AU-ROC curve analysis to complement model evaluation metrics for the context of drought stress models. In Fig 4, we can see that Random Forest-based models presented relatively high AU-ROC values (0.88) and again were higher than the values found in the LGBM (0.86), as was the case in the evaluation of cold stress models. Similarly, Random Forest consistently outperformed LightGBM in the AUPRC analysis. Random Forest obtained an AUPRC of 0.8151 compared to 0.8039 for LightGBM.

Finally, a cross-validation analysis was performed to evaluate drought stress models using the Word2Vec 3-mers set. The same 4-fold cross-validation approach was used for the Random Forest and LightGBM algorithm models. The scores obtained were:

- LightGBM: Scores – [0.72593204, 0.80840019, 0.80908555, 0.77309735]. Average of 0.78.
- Random Forest: Scores – [0.72581406, 0.79270882, 0.8020059, 0.75846608]. Average of 0.77.

In the drought stress prediction models, the prediction accuracy of the independent test set was also similar to the score values obtained from cross-validation.

### Unsupervised approach

**Fig 5:**
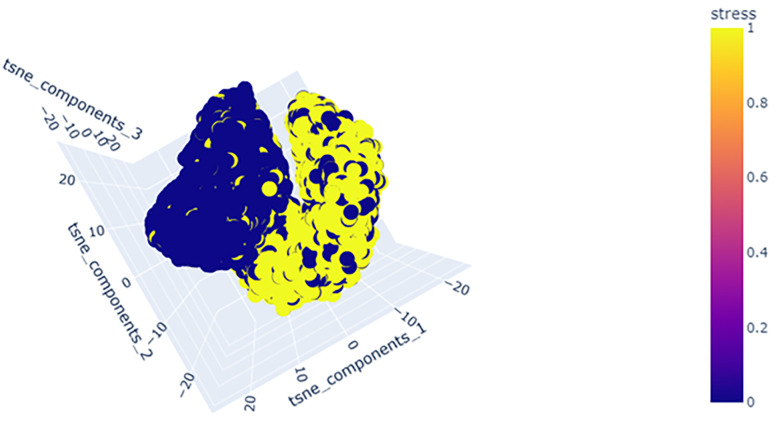
3D representation of t-SNE and components generated from Word2Vec vector compositions of circRNAs. Color range based on stress column labels (0 – control and 1 – drought stress).

In order to obtain a deeper understanding of the sequence patterns of circRNAs for predicting abiotic stress using the Word2Vec vector approach, models were also developed using unsupervised machine learning techniques. In unsupervised machine learning, the learning process aims to identify patterns within the data in order to group or organize them based on their similarities. Since we do not have labeled data in this methodology, there is no need to divide the dataset into training and test sets. In this sense, the Word2Vec vector data (based on k-mers of length 3-mers and vector size of 64) were initially grouped into 3 components using the t-SNE method, the components were colored according to the “drought stress” label and the result generated a 3D representation available in Fig 5.

In Fig 5, we can see that it was possible to segment circRNAs into 2 distinct groups according to their similarity of vector values grouped by t-SNE, even though there was some overlap in colors between the groups. This demonstrates that it is possible to predict potential involvement of circRNAs with drought stress based on their biological sequence structure and vector values. CircRNAs involved in drought stress appear to share common characteristics and patterns, which is why it was possible to cluster these data via t-SNE.

**Fig 6:**
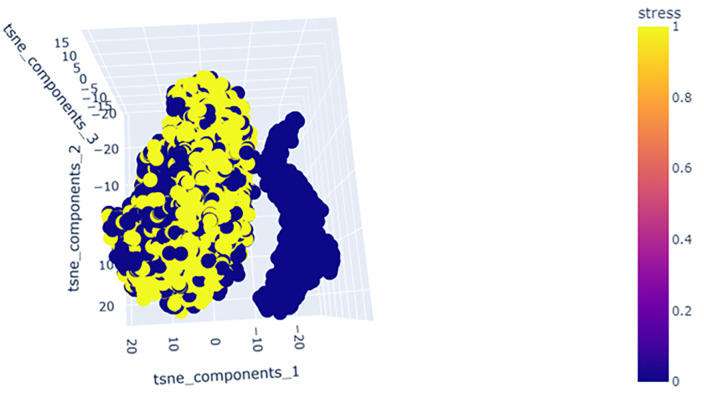
3D representation of t-SNE and components generated from Word2Vec vector compositions of circRNAs. Color range based on stress column labels (0 – control and 1 – cold stress).

In order to understand whether this behavior also applied to circRNAs belonging to the group linked to cold stress conditions, the same technique of grouping vector values into 3 components was applied using the t-SNE method based on circRNA data from the control and cold stress conditions (Fig 6).

Despite having an isolated group of circRNAs with similar characteristics belonging to the control group in the 3D representation of the components, in Fig 6 we can notice that there is more color overlap in the cold stress group than compared to what was observed in Fig 5 of the analysis of drought stress circRNAs. Perhaps because circRNAs labeled with the cold stress label share more similar patterns with circRNAs from the control condition, we can assume that this may explain the lower metrics in the binary classification models of potential involvement of circRNAs in cold stress conditions compared to the drought stress prediction models.

### Approximate nearest neighbors analysis

To understand the similarity between circRNA sequences in different biological conditions and which patterns the algorithms were learning, a nearest neighbor search was performed using Python and the Annoy algorithm. In this sense, 5724 circRNA sequences belonging to the cold stress group were sequentially analyzed and compared with sequences from cold stress and control condition and 12030 circRNA sequences belonging to the drought stress group were also analyzed and compared with sequences from drought stress and control groups to find the 5 nearest neighbors. The result is shown in Table 3.

**Table 3:** Five nearest neighbor analysis of stress-related circRNA sequences.

Data sample analyzed	Number of sequences analyzed	Number of neighbors found in the stress group	Number of neighbors found in the control group
Cold stress	5724	14617 (51.07%)	14003 (48.93%)
Drought stress	12030	44132 (73.37%)	16018 (26.63%)

These results demonstrate that in rice circRNA samples with cold stress involvement, most of the sequences of close neighbors found are also related to the cold stress group (14617 of 28620 neighbors identified – 51.07%). This suggests that the sequences of circRNAs under cold stress have a high similarity to each other, indicating possible common patterns. It is also possible to observe that the same pattern was repeated in the drought stress analysis, where circRNA sequences involved with the drought stress condition also found more similarities and neighbors that were also present in the drought stress group. A total of 73.3% of the total of neighbors were identified in circRNA sequences belonging to the drought stress group, indicating a denser network of similarity in the structure of biological sequences in this condition.

### k-mer density analysis

**Fig 7:**
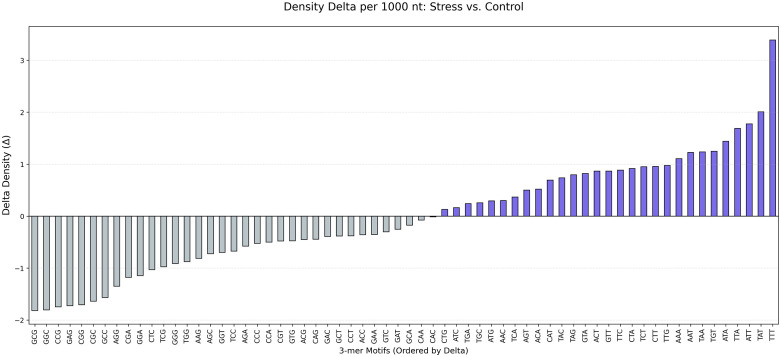
k-mer density analysis of circRNA sequences under abiotic stress conditions. Density distribution of 3-mer motifs calculated from circRNA sequences comparing the combined abiotic stress group (cold and drought) with the control condition. k-mer counts were normalized by sequence length and expressed as occurrences per 1,000 nucleotides (kb).

To further investigate whether specific sequence patterns could explain the predictive performance of the machine learning models, a k-mer density analysis was performed comparing circRNAs from control and stress conditions. The results revealed clear differences in the distribution of 3-mer motifs between control and abiotic stress groups (cold and drought), indicating that stress-responsive circRNAs exhibit distinct sequence signatures (Fig 7).

**Fig 8:**
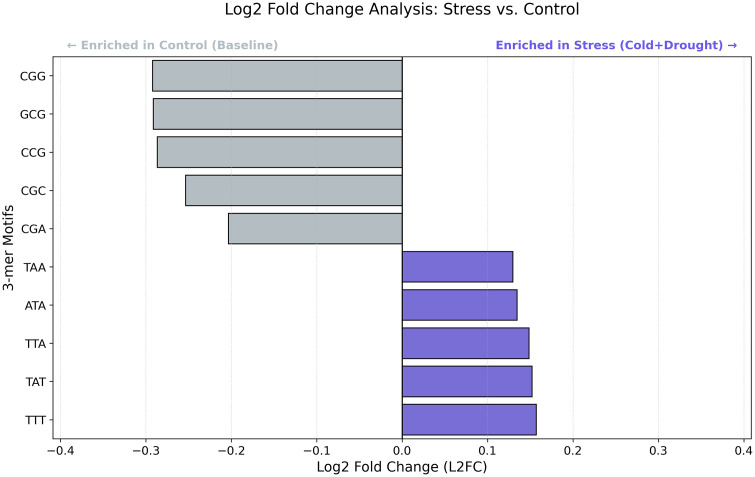
k-mer enrichment analysis based on Log2 Fold-Change. Log2 Fold-Change analysis of 3-mer motif density between stress (cold + drought) and control groups. Positive Log2FC values indicate motifs enriched in circRNAs associated with abiotic stress, whereas negative values represent motifs more frequent in the control condition.

The density analysis showed that several motifs were consistently enriched in circRNAs associated with abiotic stress conditions when compared to the control group. This enrichment became even more evident when the proportional differences between groups were evaluated using Log2 Fold-Change analysis (Fig 8). In particular, multiple motifs with higher adenine–thymine (AT) content presented positive Log2FC values, indicating that these motifs occur more frequently in circRNAs expressed under stress conditions.

A similar pattern was observed when cold and drought stress conditions were analyzed independently (S1-S4 Fig). In both scenarios, stress-associated circRNAs displayed enrichment of AT-rich 3-mer motifs relative to the control group, reinforcing the existence of characteristic sequence signatures associated with abiotic stress responses.

To further explore whether these patterns were conserved across species, additional analyses were performed separately using rice and maize circRNA datasets subjected to drought stress (S5 and S6 Figs). Although the ranking of the most enriched k-mers differed between the two species, indicating a certain degree of species-specific variability, the overall enrichment of AT-rich motifs remained consistent. Interestingly, the occurrence of these motifs was substantially higher in rice circRNAs compared to maize. For example, the 3-mer motif TTT presented a density greater than 10 occurrences per 1,000 nucleotides in rice, whereas in maize its density was slightly above 2 occurrences per 1,000 nucleotides.

### Validation using biological sequences from different plant species

To develop the circ-EnviroPredict tool, models that presented the best metrics and trained with features from Word2Vec data generated from k-mers 3-mers and vector size 64 were used. In addition, the models available in the tool were those trained with the approach based on the RandomForestClassifier algorithm. Although the metrics between the LGBMClassifier and RandomForestClassifier algorithms were very similar in the different test sets for the different conditions tested (drought and cold stress), the Random Forest-based models were more accurate in the sequences that were involved in the control condition, as demonstrated in the confusion matrices generated with the data from the test set. As in most cases, a circRNA ends up being identified and not having biological validation that it is involved with some specific stress condition, as it was possible to observe in the differences in quantities between the control set and abiotic stress sets obtained in CropCircDB, the control set being more accurate ends up being closer to the real scenarios. Therefore, leaving only the validation using the test set and validating the models with sequences obtained in other databases and articles, the models based on Random Forest made fewer mistakes, mainly in the control condition, which is why this approach was selected for implementation in the final tool.

Because machine learning models learn patterns from the available data and apply them to unseen data, one of the hypotheses of this work is that machine learning models could be able to help predict the role of circRNAs in abiotic stress conditions for different plant species (in addition to the species used in training), since circRNAs are highly conserved molecules. In this sense, the models were also validated using circRNA sequence data from the following species: *Arabidopsis thaliana, Glycine max* and *Triticum aestivum*. To perform this test, genomic sequence data, splice junctions and divergent primers of circRNAs were obtained from PlantcircBase and from four articles on circRNA identification under different environmental conditions [6,11,15,26]. Finally, these sequences were inserted into the circ-EnviroPredict tool for prediction and validation of the models (Table 4).

**Table 4:** Validation of genomic sequences, splice junctions and primers of circRNAs from *Arabidpsis thaliana, Glycine max* and *Triticum aestivum* using the circ-EnviroPredict predictor tool.

circRNA name	Organism	Plant type	Condition	Sequence type	Reference	circ-EnviroPredict probability
TGACv1_scaffold_221483_3B_circ_ig.1	*Triticum aestivum*	Monocotyledonous	drought stress	Genomic sequence	Wang et al., 2017 [6]	0.62
TRIAE_CS42_1AS_TGACv1_019693_AA0070020_circ_g.1	*Triticum aestivum*	Monocotyledonous	drought stress	Genomic sequence	Wang et al., 2017 [6]	0.63
TRIAE_CS42_3DL_TGACv1_252173_AA0888060_circ_g.1	*Triticum aestivum*	Monocotyledonous	drought stress	Genomic sequence	Wang et al., 2017 [6]	0.74
TRIAE_CS42_3DL_TGACv1_252173_AA0888060_circ_g.1	*Triticum aestivum*	Monocotyledonous	drought stress	Splice junction sequence	Wang et al., 2017 [6]	0.77
AT4G08300_circ_g.2	*Arabidpsis thaliana*	Dicotyledonous	drought stress	Genomic sequence	Zhang et al., 2019 [15]	0.53
AT4G08300_circ_g.2	*Arabidpsis thaliana*	Dicotyledonous	drought stress	Splice junction sequence	Zhang et al., 2019 [15]	0.64
AT1G03993_circ_g.1	*Arabidpsis thaliana*	Dicotyledonous	control	Genomic sequence	Chu et al., 2017 [11]	0.67
AT1G03993_circ_g.1	*Arabidpsis thaliana*	Dicotyledonous	control	Splice junction sequence	Chu et al., 2017 [11]	0.69
AT1G01060_circ_g.7	*Arabidpsis thaliana*	Dicotyledonous	control	Genomic sequence	Chu et al., 2017 [11]	0.76
AT1G01060_circ_g.7	*Arabidpsis thaliana*	Dicotyledonous	control	Splice junction sequence	Chu et al., 2017 [11]	0.80
gma_circ_0000279	*Glycine max*	Dicotyledonous	cold stress	Divergent primer	Wang et al., 2020 [26]	0.73
gma_circ_0000678	*Glycine max*	Dicotyledonous	cold stress	Divergent primer	Wang et al., 2020 [26]	0.78

The results obtained from all tests using data from different plant species (including mono and dicotyledonous species) are shown in Table 4, demonstrating that the tool was able to transpose the patterns of vector values learned via machine learning with rice and maize circRNAs to other plant species and correctly predict the different environmental conditions in which these circRNAs were in fact involved. The probability values for each sequence are available in the “circ-EnviroPredict probability” column. In addition, it was also possible to make the prediction using different types of circRNA sequences, including genomic sequences, splice junction sequences and even divergent primer sequences used to biologically validate circRNAs in the articles. This ability to transfer patterns learned via machine learning to new species may be related to the fact that circRNAs are widely distributed and conserved molecules among different plant species [4].

## Discussion

In nature, plants are constantly challenged by adverse abiotic conditions such as drought and cold. These stressors directly cause physical or chemical changes in plant cellular biomolecules, severely limiting the distribution of plants, leading to developmental alterationsand reduced crop productivity [27,28]. In this context, several research groups have been progressively publishing studies to elucidate the role and expression of circRNAs in plants under abiotic stress conditions [6,14,15], as these molecules can regulate several cellular processes by acting as miRNA sponges, as transcriptional regulators, and even by negatively modulating the expression of their parental genes [2]. Existing methods for circRNA identification, validation, and characterization of biological roles under abiotic stress conditions are often expensive and time-consuming. In addition, experimental studies may suffer from reproducibility issues. Thus, it is important to develop new computational methods that can support experimental studies and serve as alternative approaches for identifying circRNAs responsive to abiotic stress. Therefore, the present study is focused on developing a computational method using a machine learning approach to predict circRNA association with abiotic stress response based on sequence data.

Machine learning has emerged as a scalable and high-performance framework for data-driven discovery, as it can learn patterns from available data and apply them to previously unseen data [29]. Therefore, this approach can be a good alternative when compared to alignment-based tools, for example, which depend on already available biological reference sequences.

The present study demonstrate for the first time that the k-mer segmentation approach using text-to-vector transformation methodology (Word2Vec) can be used as an effective approach in generating features for machine learning models to predict the involvement of plant circRNAs under abiotic stress conditions. The Word2Vec technique has also been used in previous computational studies, such as a study published in 2020 [30] that demonstrated a method based on distributed sequence representation learning for the prediction of potential lncRNA-protein interactions and more recently, to compute semantic similarity among abiotic stresses in a study focused on predicting abiotic stress-responsive miRNA in plants [23]. However, to date, there is still no published study using this k-mer-Word2Vec approach to predict the involvement of plant circRNAs in abiotic stress conditions.

Given the successful applications of machine learning techniques for prediction involving biological sequences [24,25], different classification algorithms were evaluated in this study. The ones that presented the best metrics were the tree-based algorithms, such as LGBMClassifier, RandomForestClassifier and ExtraTreesClassifier, with particular emphasis on RandomForestClassifier. In Random Forest, multiple decision trees are created during model training. Each tree is constructed from a random subset of the training data, and by combining multiple trees, Random Forest reduces the risk of overfitting and improves generalization to new data. For the classification task, each tree casts a vote on the class for the new observation. The class that receives the most votes is the final prediction of the model [31]. The accuracy obtained for the best cold models (using an independent test dataset) through the Random Forest approach were: 0.77 in 3-mer (with vector size 64) feature set (Table 1) and 0.81 in tests of best drought models (Table 2). Although algorithms such as LGBMClassifier also presented similar metrics, Random Forest presented better performance in predicting circRNAs associated with the control condition, as demonstrated in the confusion matrices (Figs 1 and 3) and better AU-ROC metrics (Figs 2 and 4). As this scenario better reflects real biological conditions, since in general identified circRNAs are not necessarily attributed to a specific stress condition, Random Forest-based models were chosen to train and save the models available in the circ-EnviroPredict tool. Furthermore, as models trained with k-mer features of length 3-mers showed better performance, this feature set was used to train the final models available in the tool. This means that the accuracy of machine learning prediction may not necessarily improve as the length of k-mers increases.

In addition, a cross-validation analysis was also performed to evaluate machine learning models (cold and drought models) from the Word2Vec 3-mers set. The prediction accuracy of the independent test set was similar to that of the cross-validation, which showed that the developed models were not affected by overfitting or underfitting.

In order to obtain a deeper understanding of the sequence patterns of circRNAs and the correlation with abiotic stress conditions, we also performed analysis using unsupervised machine learning techniques using the t-SNE method and Annoy analysis. Using t-SNE technique, in both scenarios (cold and drought stress), it was possible to cluster the circRNA sequences into 2 distinct groups (control vs stress) (Figs 5 and 6). CircRNA sequences involved in drought stress condition appear to share common characteristics and patterns, which is why it was possible to cluster these data via t-SNE. It was also possible to cluster the circRNA sequences involved in the cold stress condition into two groups (control vs cold stress), but in this scenario there was more overlap of sequence characteristics involved with the control condition with stress conditions. In this case, it is not possible to divide the two cluster groups so separately compared to the scenario observed in the drought stress analysis.

Finally, approximate nearest neighbor analyses were also performed. The analyses obtained through the Annoy methodology demonstrated that the sequences of circRNAs under stress condition have a high similarity to each other, indicating possible common patterns and a dense network of similarity in the structure of biological sequences under an abiotic stress scenario. These patterns of sequence similarities within the same environmental condition found in the rice and maize data appear to also exist in other plant species such as: *Arabidopsis thaliana, Glycine max* and *Triticum aestivum*, as shown in Table 4. This can be explained by the fact that circRNAs are widely conserved among different plant species [4].

To further investigate the sequence patterns underlying the similarities detected by the machine learning models, an additional k-mer density analysis was performed. When circRNAs from cold and drought stress conditions were analyzed together and compared with the control group, clear differences in the distribution of 3-mer motifs were observed. Several motifs showed higher density in the stress group, and this enrichment became more evident when proportional differences were evaluated using Log2 Fold-Change analysis. In particular, multiple motifs enriched under stress conditions presented higher adenine–thymine (AT) content, suggesting that stress-responsive circRNAs may share characteristic nucleotide signatures. Such enrichment of AT-rich motifs may influence RNA structural properties and regulatory interactions, providing a biological explanation for the patterns captured by the machine learning models.

Similar trends were observed when the two stress conditions were analyzed independently. Both cold- and drought-associated circRNAs exhibited enrichment of several AT-rich 3-mer motifs relative to the control group, indicating that these sequence characteristics are not restricted to a specific stress type but may represent a broader signature associated with abiotic stress responses.

To further explore possible species-specific patterns, the drought datasets from rice and maize were analyzed separately. Although the relative ranking of the most enriched k-mers varied between species, indicating some degree of sequence variability, the overall enrichment of AT-rich motifs remained consistent. Interestingly, the abundance of these motifs was substantially higher in rice compared to maize. For example, the 3-mer motif TTT presented a density greater than 10 occurrences per 1,000 nucleotides in rice circRNAs, whereas in maize its density was slightly above 2 occurrences per 1,000 nucleotides. These observations suggest that while the exact motif composition may differ between plant species, the presence of AT-rich sequence signatures appears to be a conserved feature of circRNAs associated with abiotic stress conditions.

In recent years, increasing attention has been given to interpretability and feature visualization in machine learning models applied to biological data. Several recent studies have highlighted the importance of exploring learned feature representations to better understand how predictive models capture biologically meaningful patterns. For example, deep learning frameworks have been combined with feature visualization strategies to analyze fused representations of biological entities and improve model interpretability in complex prediction tasks [32,33]. Inspired by this perspective, in the present study we combined embedding-based sequence representations with visualization and compositional analyses in order to better understand the patterns learned by the predictive models. In particular, the t-SNE projection of sequence embeddings together with the k-mer density analysis allowed us to explore sequence composition differences between control and abiotic stress conditions, providing additional biological insight into the sequence signatures captured by the machine learning framework.

Together, these findings reinforce that stress-responsive circRNAs possess identifiable sequence patterns that can be captured through k-mer-based representations, providing additional biological interpretability to the predictive models developed in this study.

In recent years, several computational approaches have been proposed to predict circRNA-related biological functions using different types of biological data and machine learning strategies. For example, the model proposed in LDA-VGHB integrates topic modeling and graph-based learning to infer circRNA–disease associations using heterogeneous biological networks and similarity matrices [34]. Similarly, deep learning frameworks such as GEnDDn employ hybrid neural architectures combining convolutional and recurrent neural networks to capture complex sequence patterns and predict molecular interactions involving circRNAs [35]. Although these approaches have demonstrated strong predictive performance, they generally rely on large datasets, complex architectures, or extensive biological annotation. In contrast, the methodology proposed in the present study adopts a lightweight sequence-based representation using k-mer segmentation combined with Word2Vec embeddings and tree-based machine learning algorithms. This strategy enables efficient feature extraction while maintaining competitive predictive performance and allowing easier application to plant species with limited functional annotation.

Although several computational approaches have been developed for circRNA functional prediction, only a limited number of studies have specifically addressed abiotic stress-responsive circRNAs in plants. Until the present moment, only one similar study has been published using circRNA sequence data and abiotic stress prediction by machine learning. In this study published in 2024, researchers developed a machine learning tool (AScirRNA) with the aim of discovering abiotic stress-responsive circRNAs in plant genome using K-tuple nucleotide and Pseudo KNC as features [25]. Here, we use a different approach, based on features generated by Word2Vec technique to predict the potential association between circRNAs and specific types of abiotic stress – cold and drought. In the study published by Pradhan et al. [25], the researchers also found the best metrics using tree-based algorithms, but in their case the XGBoost algorithm presented the best metrics for predicting abiotic stress in general (without distinction of specific models for cold and drought stress). Using an independent test dataset, Pradhan et al. obtained in the developed models an accuracy of 73.13% for KNC feature set and 73.52% for PKNC feature set, respectively. Our results using the k-mers approach based on Word2Vec vectors as features achieved similar performance in the models developed for cold stress prediction (77%) and higher metrics in drought stress models (81%). Together, these two works help to confirm that it is possible to predict the involvement of circRNAs in abiotic stress conditions using biological sequence data as features.

## Conclusion

All these results indicate that it is possible to classify circRNAs associated with environmental stress conditions using only sequence-derived features and machine learning techniques. Although the developed models did not reach extremely high accuracy values, they can still be useful as preliminary screening tools or as in silico validation steps to complement experimental studies. These studies are extremely innovative and have great potential.

In addition to the predictive models, complementary analyses such as sequence embedding visualization and k-mer density analysis provided further insight into the sequence characteristics associated with stress-responsive circRNAs. In particular, the enrichment of specific AT-rich 3-mer motifs observed in circRNAs under abiotic stress conditions suggests that these molecules may share characteristic sequence signatures that are captured by the machine learning models. Such compositional patterns help to explain the predictive capacity of the models and provide additional biological interpretability to the proposed framework.

The code and datasets for this study are publicly available at https://github.com/mariacmartins/circ-enviropredict/. In addition, the circ-EnviroPredict tool (available online at: https://circ-enviropredict.streamlit.app/), offers researchers the possibility of predicting the potential involvement of circRNAs molecules in abiotic stress conditions through a simple and user-friendly interface. The final models implemented in the circ-EnviroPredict tool, based on the Random Forest algorithm, achieved consistent performance across multiple evaluation metrics. For cold stress prediction, the model reached an accuracy of 0.77, precision of 0.76, recall of 0.75, F1-score of 0.75, AU-ROC of 0.82, and AUPRC of 0.76. For drought stress prediction, the model achieved an accuracy of 0.81, precision of 0.81, recall of 0.81, F1-score of 0.81, AU-ROC of 0.88, and AUPRC of 0.82.

This tool may serve as a complementary computational resource to support experimental studies in plant stress biology. In future work, the predictive models may be further improved by incorporating additional circRNA datasets from different plant species and environmental conditions, which could enrich the training sets and potentially increase the predictive performance and generalization capacity of the models.

## Supporting information

S1 Table Comparison of model performance across different k-mer lengths (k = 3, 4, and 5). No improvement was observed for larger k-mer sizes, while computational cost increased, supporting the use of k = 3 in the final models.
(DOCX)

S1 Fig k-mer density analysis of circRNA sequences under cold stress conditions. Density distribution of 3-mer motifs calculated from circRNA sequences comparing the cold stress group with the control condition. k-mer counts were normalized by sequence length and expressed as occurrences per 1,000 nucleotides (kb).
(TIFF)

S2 Fig k-mer enrichment analysis based on Log2 Fold-Change. Log2 Fold-Change analysis of 3-mer motif density between cold stress and control groups. Positive Log2FC values indicate motifs enriched in circRNAs associated with cold stress, whereas negative values represent motifs more frequent in the control condition.
(TIFF)

S3 Fig k-mer density analysis of circRNA sequences under drought stress conditions. Density distribution of 3-mer motifs calculated from circRNA sequences comparing the drought stress group with the control condition. k-mer counts were normalized by sequence length and expressed as occurrences per 1,000 nucleotides (kb).
(TIFF)

S4 Fig k-mer enrichment analysis based on Log2 Fold-Change. Log2 Fold-Change analysis of 3-mer motif density between drought stress and control groups. Positive Log2FC values indicate motifs enriched in circRNAs associated with drought stress, whereas negative values represent motifs more frequent in the control condition.
(TIFF)

S5 Fig k-mer density analysis of circRNA sequences from rice under drought stress conditions. Density distribution of 3-mer motifs calculated from circRNA sequences comparing the drought stress group with the control condition. k-mer counts were normalized by sequence length and expressed as occurrences per 1,000 nucleotides (kb).
(TIFF)

S6 Fig k-mer density analysis of circRNA sequences from maize under drought stress conditions. Density distribution of 3-mer motifs calculated from circRNA sequences comparing the drought stress group with the control condition. k-mer counts were normalized by sequence length and expressed as occurrences per 1,000 nucleotides (kb).
(TIFF)
