# Supplementary material for: Circ-EnviroPredict: A machine learning-based tool to predict potential involvement of circRNAs with cold and drought stress through a Word2Vec approach
Source: PLoS One. 2026 Jun 18;21(6):e0350943. doi: 10.1371/journal.pone.0350943 (PMC13278450; doi:10.1371/journal.pone.0350943)
Supplement: S1 Table — No improvement was observed for larger k-mer sizes, while computational cost increased, supporting the use of k = 3 in the final models. (DOCX) [file pone.0350943.s001.docx]

| Model | Feature Set | Accuracy | Precision | Recall | F1-score |
| --- | --- | --- | --- | --- | --- |
| RandomForestClassifier (Cold Model) | 3-mers  (vector size 64) | 0.77 | 0.76 | 0.75 | 0.75 |
|  | 4-mers  (vector size 64) | 0.72 | 0.71 | 0.70 | 0.70 |
|  | 5-mers  (vector size 64) | 0.72 | 0.71 | 0.70 | 0.70 |
| RandomForestClassifier (Drought Model) | 3-mers  (vector size 64) | 0.81 | 0.81 | 0.81 | 0.81 |
|  | 4-mers  (vector size 64) | 0.80 | 0.80 | 0.80 | 0.80 |
|  | 5-mers  (vector size 64) | 0.80 | 0.80 | 0.80 | 0.80 |
