# Supplementary figures and images for: Circ-EnviroPredict: A machine learning-based tool to predict potential involvement of circRNAs with cold and drought stress through a Word2Vec approach
Source: PLoS One. 2026 Jun 18;21(6):e0350943. doi: 10.1371/journal.pone.0350943 (PMC13278450; doi:10.1371/journal.pone.0350943)

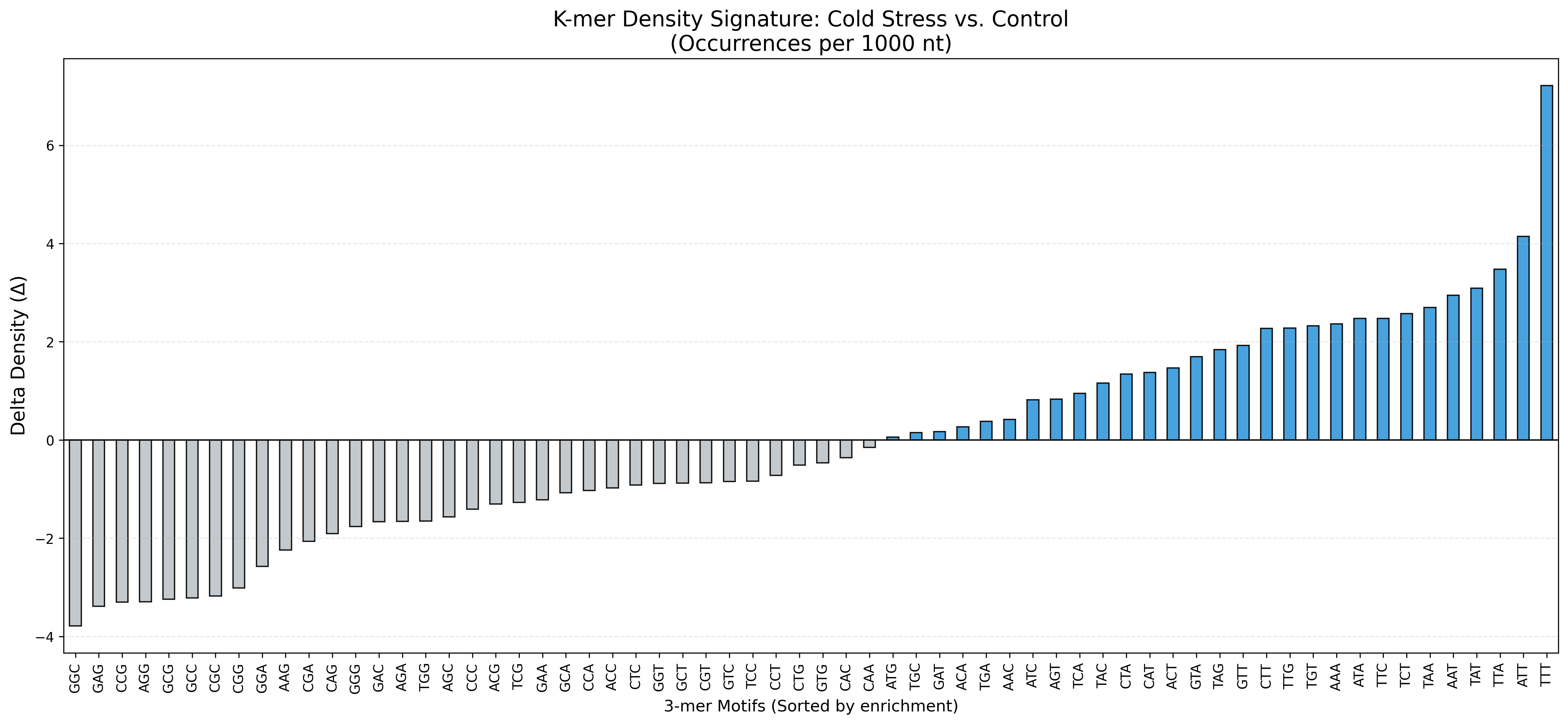

Supplement: S1 Fig — Density distribution of 3-mer motifs calculated from circRNA sequences comparing the cold stress group with the control condition. k-mer counts were normalized by sequence length and expressed as occurrences per 1,000 nucleotides (kb). (TIFF) [file pone.0350943.s002.tiff]

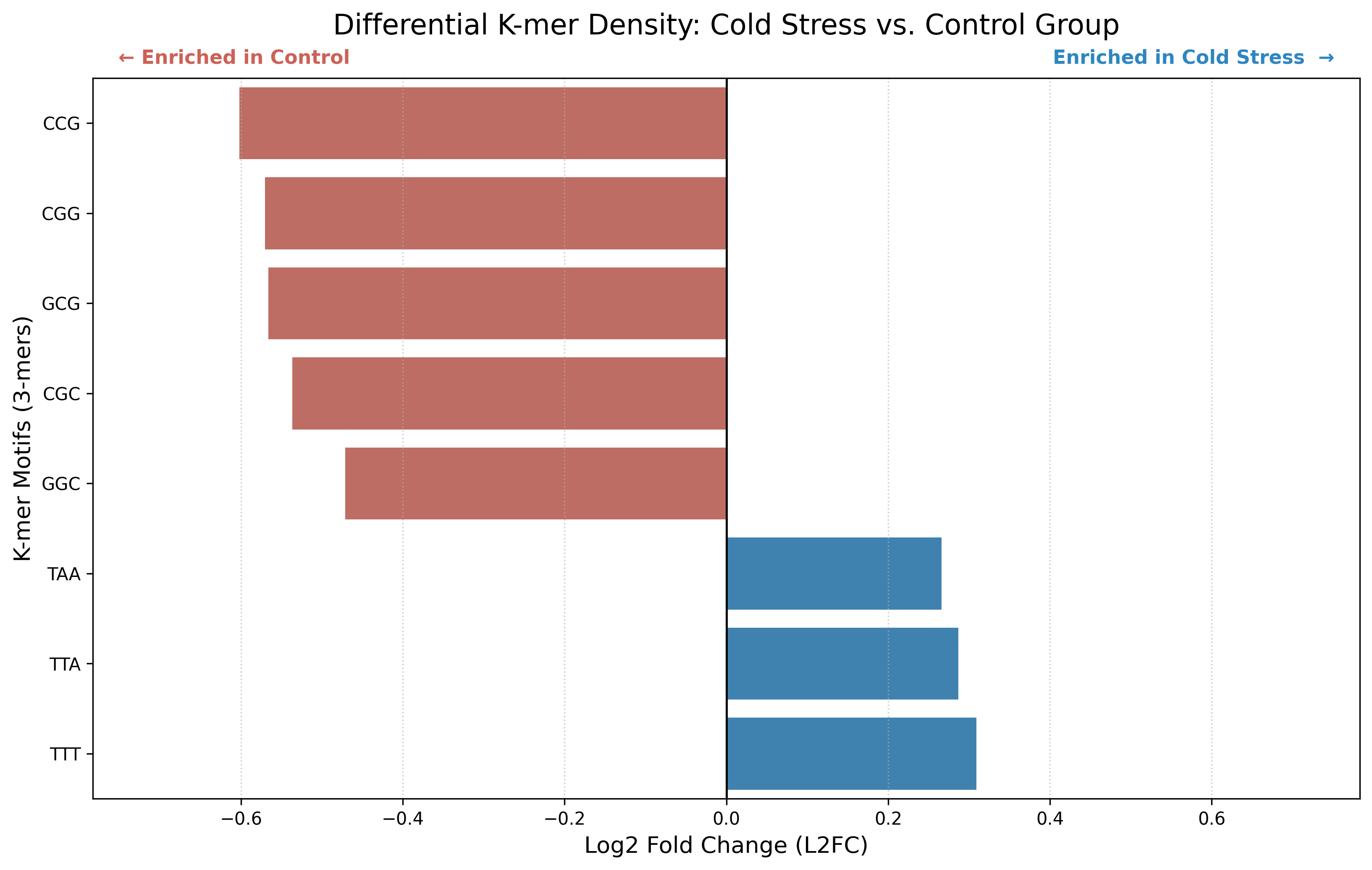

Supplement: S2 Fig — Log2 Fold-Change analysis of 3-mer motif density between cold stress and control groups. Positive Log2FC values indicate motifs enriched in circRNAs associated with cold stress, whereas negative values represent motifs more frequent in the control condition. (TIFF) [file pone.0350943.s003.tiff]

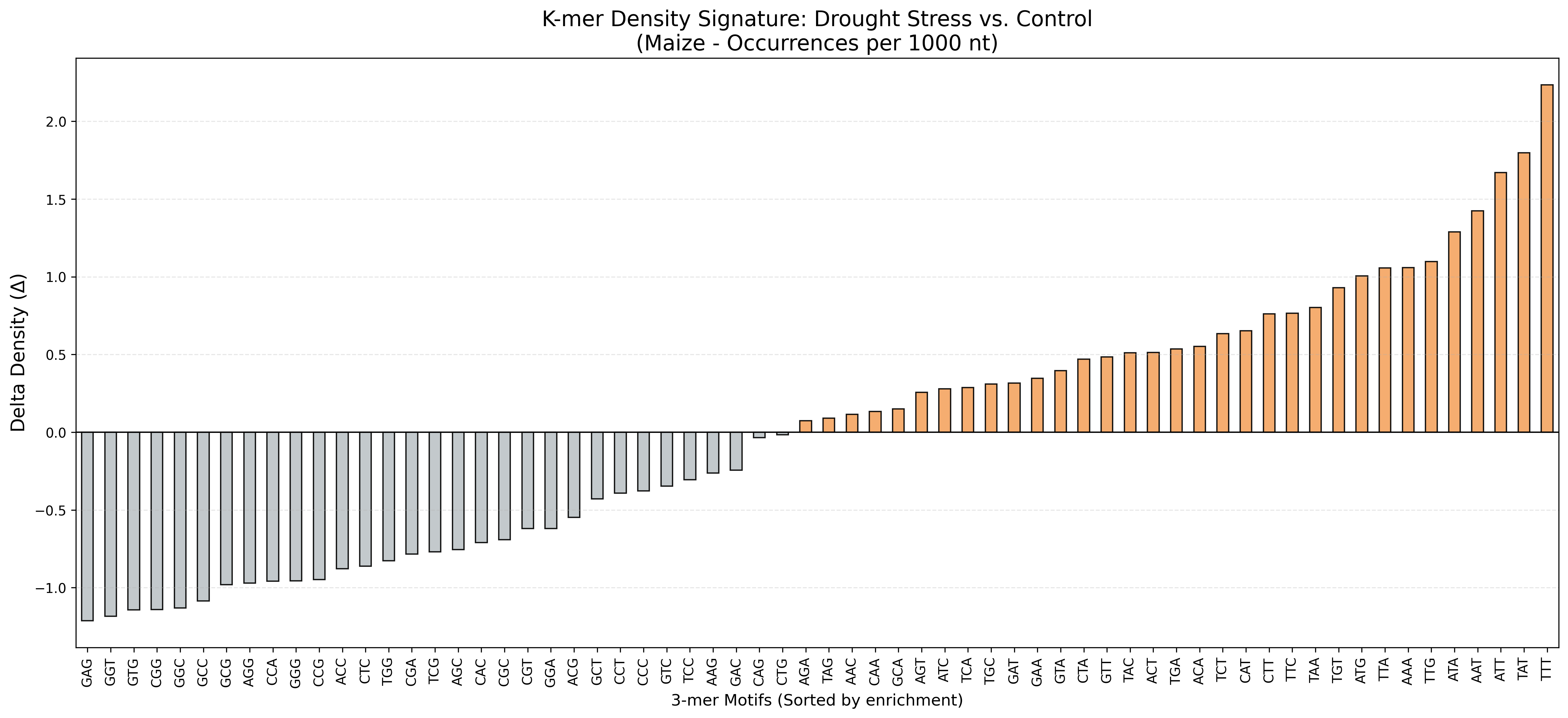

Supplement: S3 Fig — Density distribution of 3-mer motifs calculated from circRNA sequences comparing the drought stress group with the control condition. k-mer counts were normalized by sequence length and expressed as occurrences per 1,000 nucleotides (kb). (TIFF) [file pone.0350943.s004.tiff]

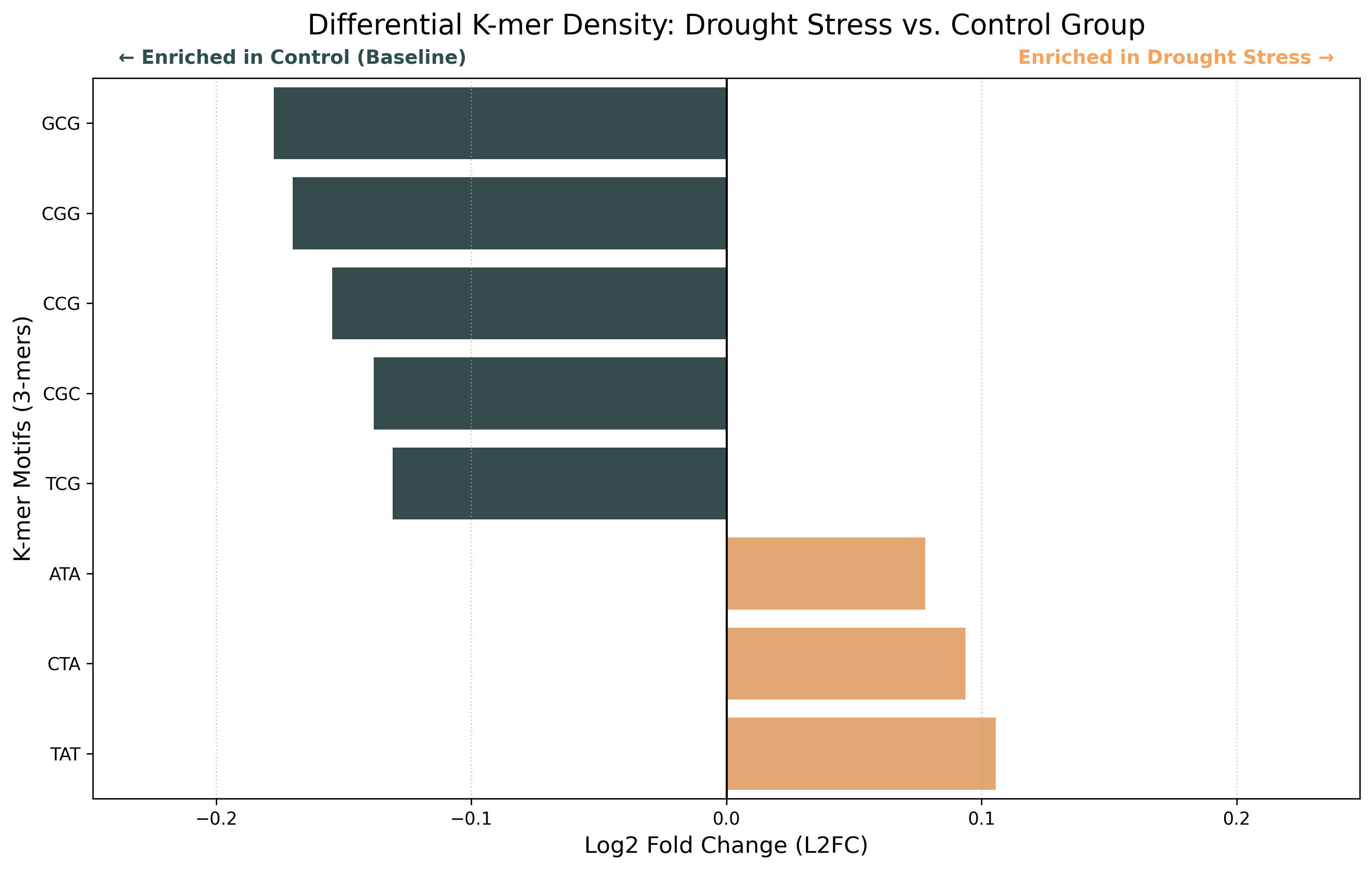

Supplement: S4 Fig — Log2 Fold-Change analysis of 3-mer motif density between drought stress and control groups. Positive Log2FC values indicate motifs enriched in circRNAs associated with drought stress, whereas negative values represent motifs more frequent in the control condition. (TIFF) [file pone.0350943.s005.tiff]

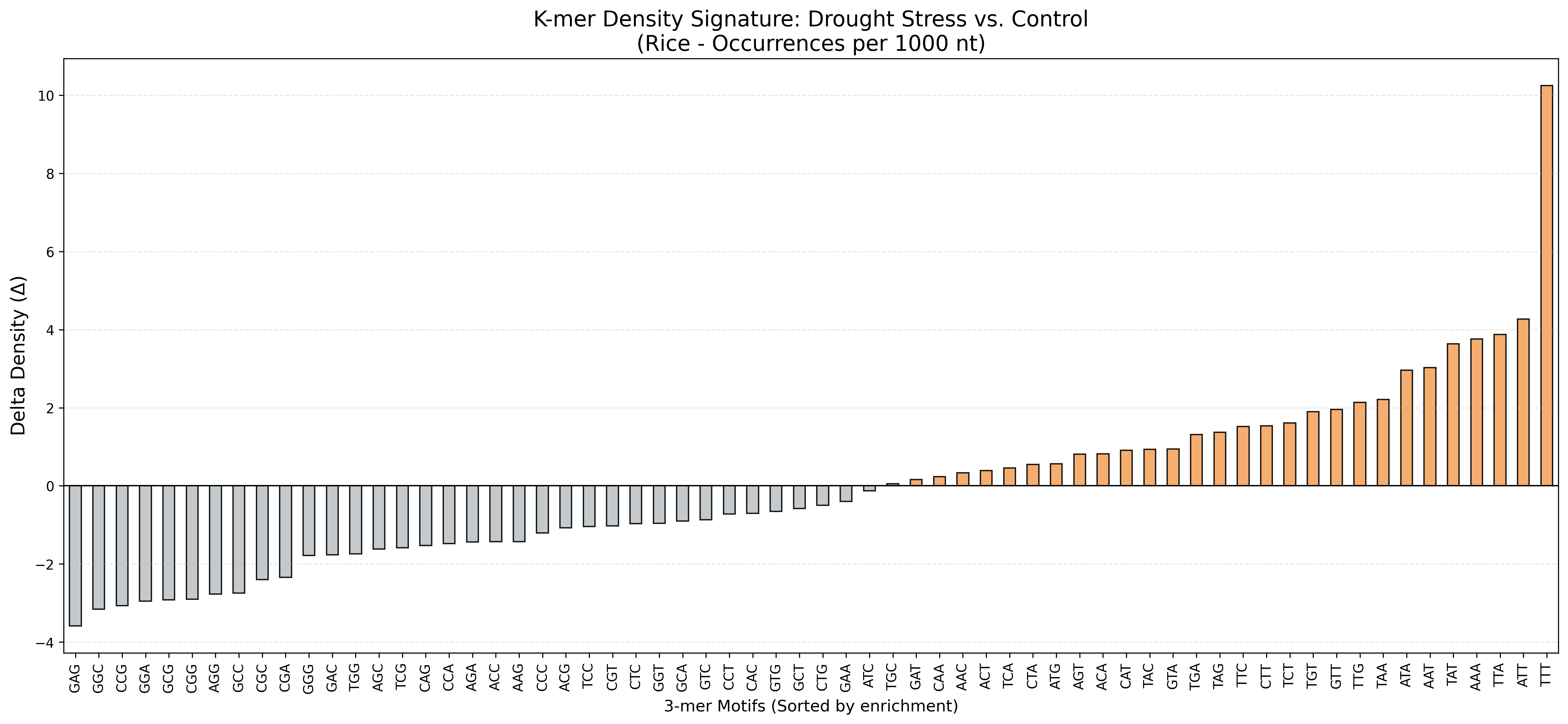

Supplement: S5 Fig — Density distribution of 3-mer motifs calculated from circRNA sequences comparing the drought stress group with the control condition. k-mer counts were normalized by sequence length and expressed as occurrences per 1,000 nucleotides (kb). (TIFF) [file pone.0350943.s006.tiff]

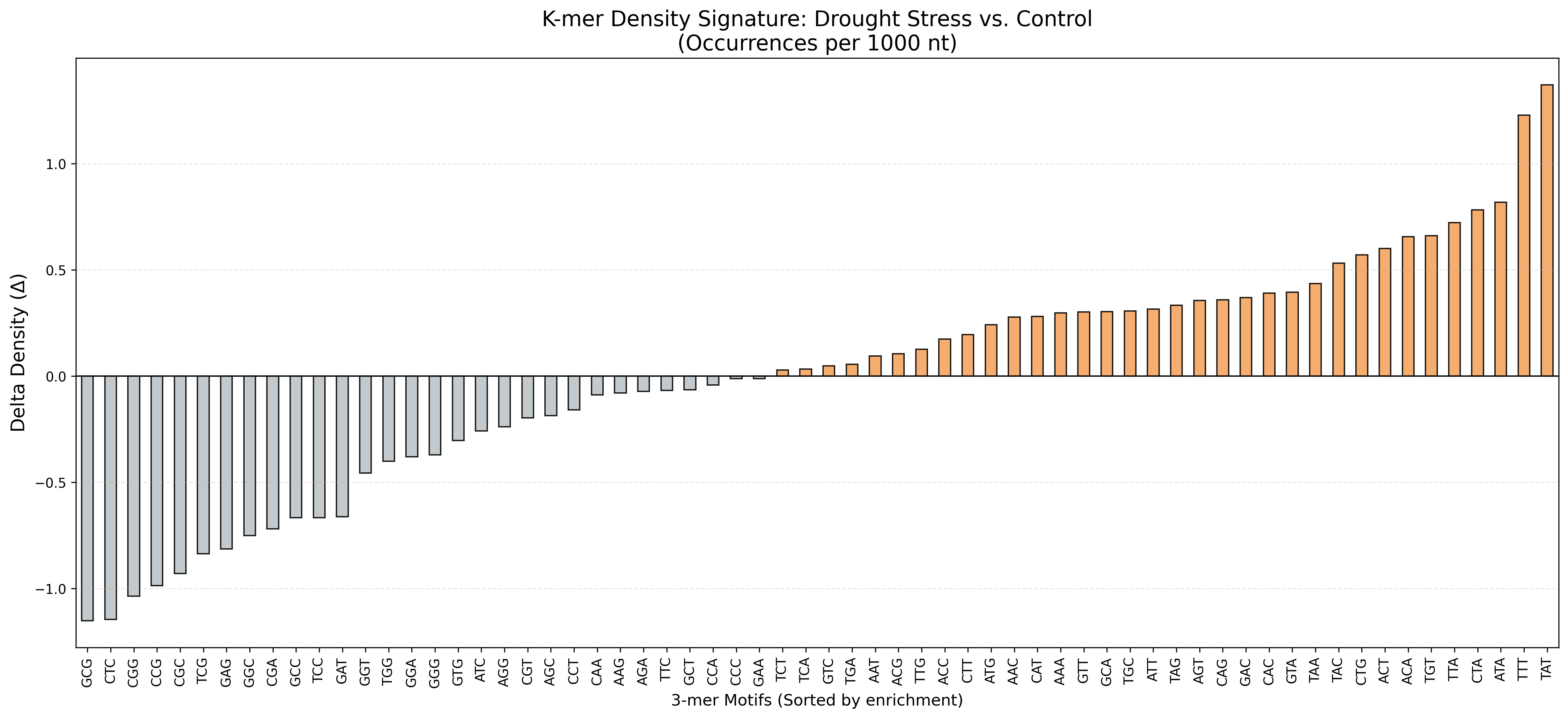

Supplement: S6 Fig — Density distribution of 3-mer motifs calculated from circRNA sequences comparing the drought stress group with the control condition. k-mer counts were normalized by sequence length and expressed as occurrences per 1,000 nucleotides (kb). (TIFF) [file pone.0350943.s007.tiff]
